# Immune Status of Pancreatic Cancer Patients Receiving Cryosurgery

**DOI:** 10.3390/medsci7060073

**Published:** 2019-06-22

**Authors:** Vladimir M. Zemskov, Konstantin N. Pronko, Dmitrii A. Ionkin, Alexei V. Chzhao, Maria N. Kozlova, Alexander A. Barsukov, Nadezhda S. Shishkina, Valentina S. Demidova, Andrei M. Zemskov, Amiran Sh. Revishvili

**Affiliations:** 1 A.V. Vishnevsky National Medical Research Center of Surgery, Moscow 117997, Russia; ionkin@ixv.ru (D.A.I.); chzhao@ixv.ru (A.V.C.); kozlova@ixv.ru (M.N.K.); barsukov@ixv.ru (A.A.B.); nadya-vesy@mail.ru (N.S.S.); demidova@ixv.ru (V.S.D.); amirevi@mail.ru (A.S.R.); 2 Department of Research and Development at Facecontrol, Inc., Miami, FL 33131, USA; k.pronko@facecontrol.biz; 3 Department of Microbiology of Burdenko, Voronezh State Medical University, Voronezh 394000, Russia; zemskov@vsmaburdenko.ru

**Keywords:** pancreatic cancer, cryosurgery, immune status

## Abstract

Cryosurgery used on patients with unresectable pancreatic cancer improved their quality of life, but mainly because of the pain relief. In postoperative patients, multifaceted changes in immunity were found, and the state of the immune system prior to surgery often was a decisive factor to indicate whether further disorders in the postoperative period would develop, or by contrast, it would boost its recovery. Some patients receiving cryosurgery showed immune system imbalance and activation, and of antitumor immunity in particular. It has been suggested that the advisability of immunotropic therapy for specific treatment algorithms should be predicted or the therapy should be suspended at some pathologic stage, and this has been immunologically confirmed. Cryosurgery should be considered as a reasonable alternative to the existing types of surgery for pancreatic cancer or as an essential component of multimodal therapy, consisting of topical cryosurgery, chemotherapy, and immunotropic therapy, to boost antitumor immunity and to discontinue cytoreductive therapy due to its toxic effects.

## 1. Introduction

It has been established that pancreatic cancer patients have the worst treatment outcome of all significant cancer patients, both in Russia and other countries around the world [1,2,3]. The conservative surgical resection represents the primary type of pancreatic cancer treatment, which alone or in combination with chemotherapy, radiation therapy, and other treatment options provides low survival rates [2,3,4]. With the development and clinical use of cryosurgery for pancreatic cancer, the treatment outcomes for this group of patients have been significantly improved, such as survival rate and quality of life [2,3,4,5]. To date, the primary focus of medical research is the development of minimally invasive surgery procedures based on physical factors of intervention. As the specific nature of freezing of the living tissues comes to be better understood, and instrumental methods of analysis have become more sophisticated, cryosurgery has turned out to be more competitive versus conventional surgery, and it belongs rightfully to the most advanced healthcare technologies as a relatively safe and organ-preserving method [6]. 

The basic principle involved in cryosurgery consists of in-situ exposure of pathologically changed organs and tissues to freezing (firstly, for destruction) [7,8]. The aim of this technique is not only to destroy cancer cells but also to activate self-regulation mechanisms among all the body systems—in metabolism and endocrinology, mental health, and to induce the functional reserve capacity of the protective immunity, and antitumor immunity in particular [9]. Moreover, cryosurgery is a less invasive procedure that activates reparative processes and has a marked anesthetic and local hemostatic effect [5,10].

The immune system mobilizes a very early response to surgery, infection, and other kinds of exposure, providing a possibility to predict the development of different complications. It enables the justified use of targeted therapy, including the use of immune modulation, where the target cells are destroyed, allowing progress in therapy to be tracked. Cryosurgery is known to activate the immune system surveillance and antitumor immune response, while in other patients these immunological parameters can be concomitantly suppressed [11,12]. Based on these data it is possible to create the prediction models and algorithm for chemotherapy or discontinue its use at some treatment stage depending on the state of the immune system of a patient. The rationale for the combined use of chemotherapy and immunotherapy drugs is that it increases anti-cancer therapy resistance and capacity to eliminate tumors, as well as helps manage the toxic side effects of chemotherapy drugs.

The objective of the present prospective study was to evaluate the state of the immune system in pancreatic cancer patients exposed to localized cryosurgery to assign further schemes and algorithms for chemotherapy and immunotherapy and to assess the efficacy of their combined use.

## 2. Materials and Methods

Clinical and laboratory studies and immunological tests were conducted in 18 pancreatic cancer patients, while the control group was composed of 20 healthy volunteers. The timeframes of the research study were as follows: before cryosurgery, the first day after cryosurgery, on day 3 to 5 after cryosurgery, and day 7 to 10 after cryosurgery. In some cases, remote measurements were taken in 3 months, at 1.7 years, and 2.8 years after cryosurgery.

Phenotypic analysis of immunocompetent and phagocytic cells (neutrophils and monocytes) was performed without any additional cellular activation by flow cytometry with monoclonal antibodies (monABs) (BD Biosciences, Becton, Dickinson, San Joe, CA, USA). The cellular phenotypes and the CD4+/CD8+ ratio (low ratio <1 was associated with the development of severe inflammation, often in response to bacterial infection, and with enhanced migration of T-lymphocytes to tumor sites) were calculated on a BD FACS ( Fluorescence Activated Cell Sorter) Calibur according to the manufacturer’s recommendations, with 50 μL of whole human blood with ethylenediaminetetraacetate (EDTA) and its subsequent treatment with monABs (CD3, CD4, CD8, CD16, CD21, CD25, СD4+СD25+, CD14, CD11b, СD45, CD54, CD56, CD64, CD70, CD95, HLA–DR/CD3, CD56+CD16+, CD56+CD16− CD3+CD56+), which were labeled with fluorescein isothiocyanate (FITC) or phycoerythrin (PE) or FITC/PE-labeled; erythrocytes were removed by a lysis buffer. To identify granulocytes or monocytes expressing different FITC-labeled markers, they were additionally stained with their respective PE-labeled monABs—CD66b or CD14. The shift indices (ShIs) of blood lymphocytes (ShIBL) and the leukocytic index of intoxication (LII). The state of immune exhaustion (as well as suppression of its functional activity) was also determined using the corresponding immune markers—it developed when T lymphocyte ratios CD11b+\CD25+ (↑↑) and CD11b+\HLA-DR+ (↑↑) showed the statistically significant increase in number above the benchmark points and when CD25+\HLA-DR+ (↓↓) ratio decreased in number.

The analysis of three classes of immunoglobulins was performed with the monospecific antisera produced by Sinteko (Moscow, Russia), the method of turbidimetry, and a semiautomated biochemical Screen Master Plus analyzer (Hospitex Diagnostics S. A., Sesto Florentino, Italy) at a wavelength of 340 nm. Oxygen metabolism in phagocytic cells was analyzed (on a multiple Synergy 2 SLAD detector-analyzer, Winooski, Vermont, USA) with chemiluminescence (CL), adding luminol or lucigenin used to detect generated reactive oxygen species (ROS) to enhance the chemiluminescence reaction. According to some researchers, it is essential that the oxidation of lucigenin mainly helps identify the extracellular generation of superoxide radical, whereas luminol oxidation mainly identifies intracellular generation [13,14]. The reaction was conducted in wells using 150 μL luminol or lucigenin solution, 25 μL normal human serum (opsonized) zymosan suspension, and 5 μL whole blood of patients. Four repeated blood samples were used. The blood was extracted into standard tubes with EDTA, adhered on walls, and preserved before reaction at room temperature. First, the device was warmed up to the working condition under the standard program of analysis; when the temperature reached 37 °C, the blood samples were dissolved with initial mixing in four repeated plate wells for each patient before the plate was placed into the device. After that, the plate was placed into the device, and the CL (chemiluminescence) was analyzed for 50 min with 200-fold enhancement of the signal and a minimal interval of reading and shaking before each analysis in automatic mode. In the analysis, we used 96-well, flat-bottom, polystyrol plates.

When performing the statistical analysis only the statistically significant differences determining the degree of immune insufficiency or stimulation were used in all cases to construct graphs to analyze the cell count with different immune markers in patients. We had previously developed a method for the reliability analysis of these degrees [15]. The magnitude of changes in individual parameters was calculated using the immune disorder formula (IDF): (patient score/donor score- 1) × 100%. Changes in parameter values of up to 33% indicate a minor degree (I- non significant, *P* > 0.05) of the immune disorder, changes of 33% to 66% imply a significant disorder (II- significant disorder, *P* ≤ 0.01), and changes exceeding 66% showed severe disorders (severe immune disorder *P* ≤ 0.001).

The work used cryodestruction methods that have been used since the middle of the last century in all developed countries for the treatment of tumors of different localization and do not require any permits or consents. In this paper, we study the reactions of various systems of the body in the process of treating tumors, which allows us to expand our understanding of the indirect influence of cryomethods on various aspects of the pathophysiology of tumors in the process of local exposure and to determine changes in the immune system of the body. This study is not a clinical trial. Therefore, all CONSORT 2010 items do not require a response, since the studies were carried out within the framework of permitted surgical and immune methods. Sponsors did not participate in the design, execution, interpretation, or writing of the research or writing. All subjects gave their informed consent to inclusion before they participated in the study. All clinical, laboratory and immune studies were carried out in accordance with the ethical standards of the Helsinki Declaration.The study protocol contained ethical aspects and information on how the principles of the Helsinki Declaration are ensured.

## 3. Results and Discussion

**Figure 1:**
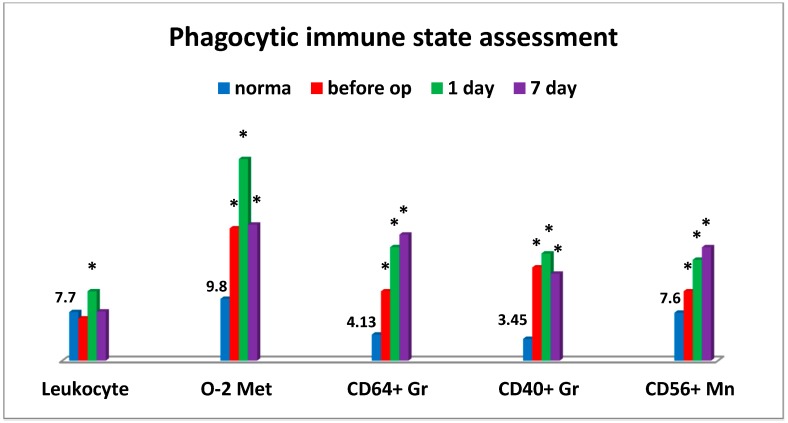
Evaluation of phagocytic immune unit. Legend: O-2 Met = oxygenic metabolism; conditional unit/1000 Nph = neutrophils; % Gr = granulocytes; Mn = monocytes; Leukocyte = billion/L; op = operation; * significance of differences between groups in all figures are reliable when *P* < 0.01 or *P* < 0.001.

An interesting phenomenon we have encountered is that the significant changes in the critical parameters of the immune system were observed in pancreatic cancer patients already at the preoperative stage (Figure 1). More than half of patients had a significantly enhanced oxygen metabolism in phagocytic cells generating intracellular ROS. Alongside with this, the relative level of granulocyte counts responsible for expression of a high-affinity Fcγ Receptor (CD64) was accumulated. Besides the levels of similar cell counts expressing tumor necrosis factor-alpha (TNF-α) superfamily member 5 (CD40) and monocytes expressing NCAM-1 (CD56), adhesion molecules increased as well. The latter could be indicative of inflammatory tissue-damaging processes that were likely to be induced by the increased accumulation of reactive oxygen species in tissues. At the same time, it is beyond argument that all changes in monocytic and granulocytic cells were indicative of their significant activation (Figure 1).

**Figure 2:**
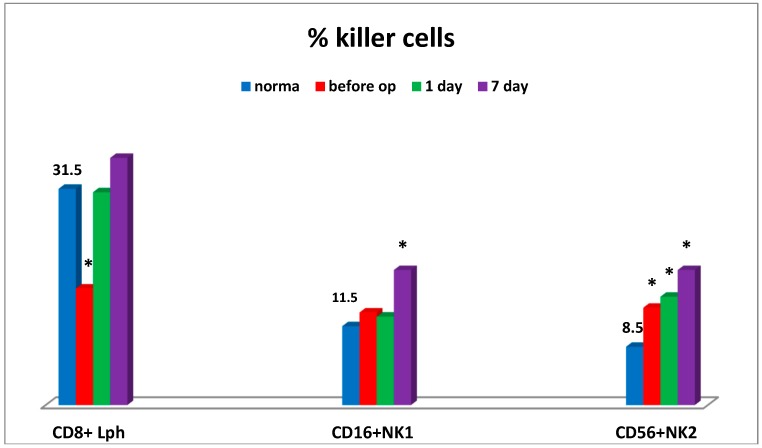
Dynamics of the content of killer cells in patients. Legend: Lph = lymphocytes; NK1 = natural killer cells; NK2 = natural killer cells; * significance of differences; op = operation.

Moreover, the sharp decrease in CD4+/CD8+ ratio was also observed at the preoperative stage in connection with humoral immune deficiency (IgM), thus being indicative of the evident inflammation state and enhanced migration of T-lymphocytes to tumor sites. Severe cytotoxic T lymphocyte deficiency and elevated levels of natural killer cells No. 2 (CD56) were found at the preoperative stage. At that stage, the levels of natural killer cells No. 1 (CD16) (Figure 2), natural killer cells as effector cells (CD56+CD16+), and natural killer cells as regulator cells (CD56+CD16-) were sufficient (not shown).

Apparently, it may be indicative of some NK cellular imbalance in tumor-bearing patients. The use of cryosurgery resulted in specific changes in the immune system, mainly manifested by its activation (Figure 1). Thus, on day 1 after cryosurgery, the vast majority of patients were found to have a predominant increase in the number of white blood cells with a left shift, granulocytic cells expressing TNF-α analogues (CD40+) and high-affinity IgG receptors (CD64) that could be indicative of functional activation of phagocytic cells. During all postoperative period, the number of patients with the enhanced oxidative stress remained unchanged (within 60%) as compared to the baseline information. On day 1 to 7 post operation a significant (up to 55%) increase in the number of patients with inflammatory tissue-damaging processes (CD56+ monocytes) was observed, which was only slightly reduced (to 40%) on the day of discharge (not shown in the table). It should be noted that abovementioned accumulation of high amounts of reactive oxygen species produced as a result of enhanced phagocyte-derived oxidative stress could be a tissue-damaging factor.

On day 7 some patients showed the apparent increase in the natural killer cell potential (cytotoxic T lymphocytes, NK cells) (Figure 2), which may be associated with the activation of anti-tumor immunity at the NK cell system level. 

**Figure 3:**
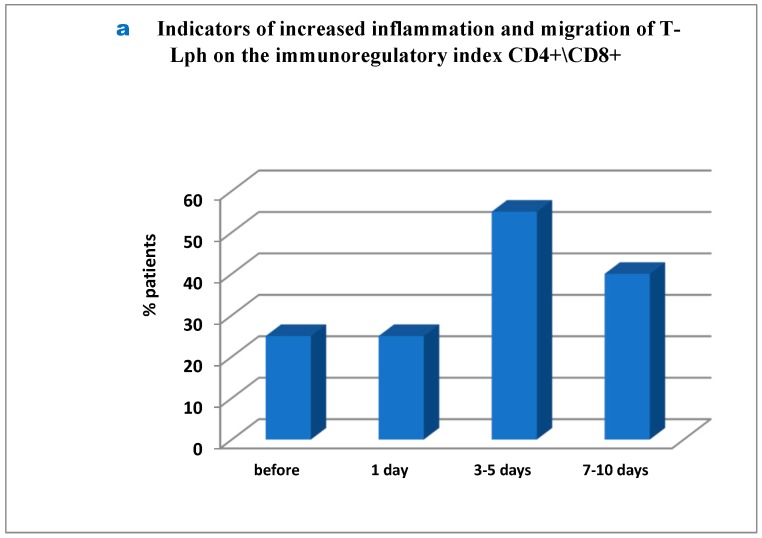

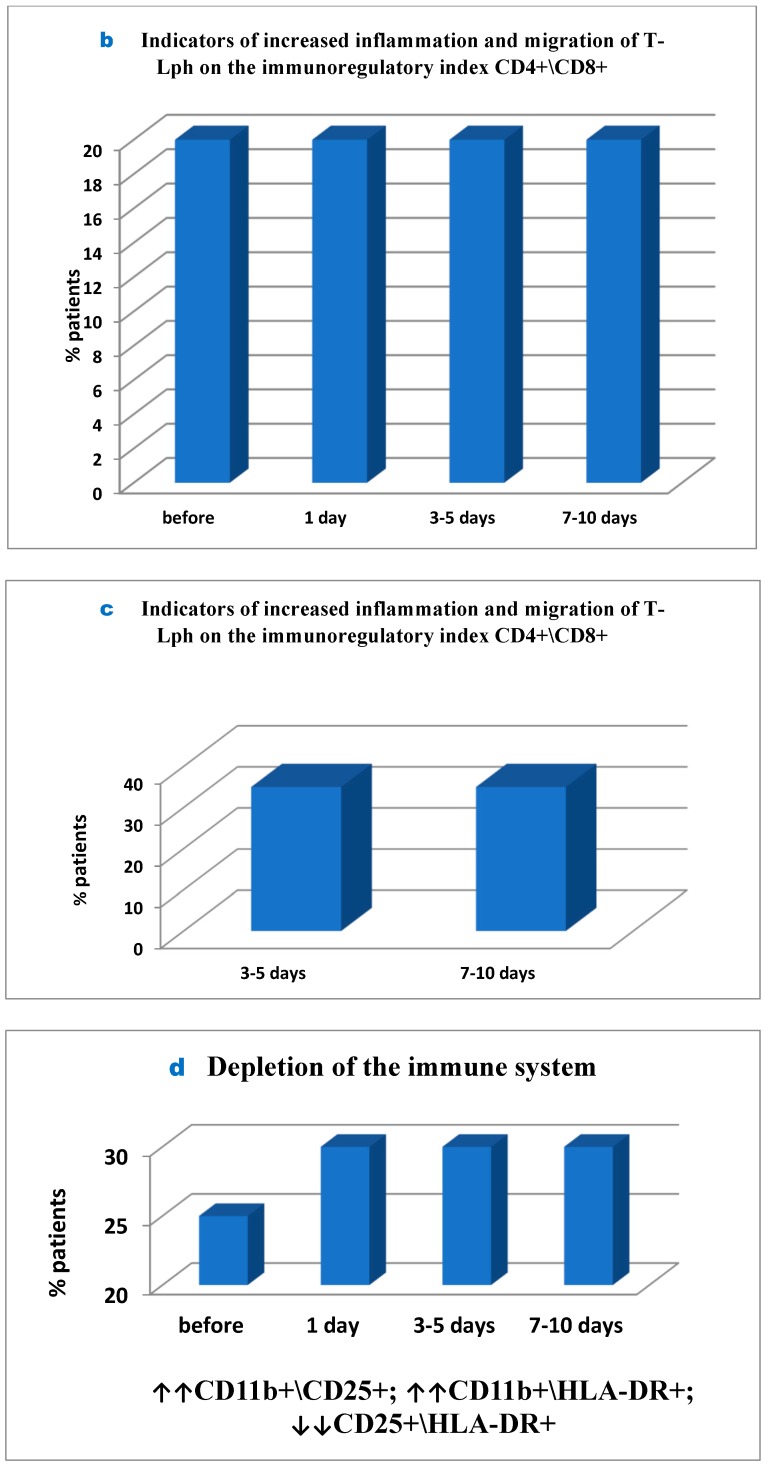

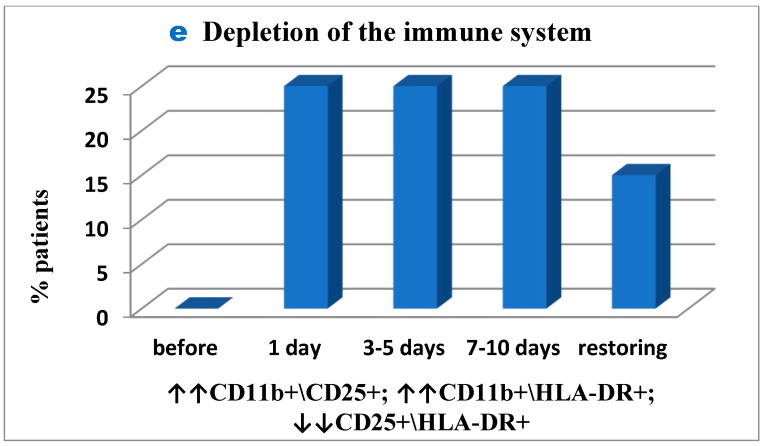
Indicators of enhanced inflammation and migration of T-lymphocytes in patients after cryo-destruction. Legend: T-Lph = T-lymphocytes; number of days = after operation. Patient depletion of the immune system—evaluation performed to raise and lower immune indexes) were recorded prior to surgery.

Interesting but so far controversial findings were obtained in the analysis of several immune ratios responsible for basic mechanisms used in the immune system. First, it was found that before surgery as much as 25% of patients showed severe inflammation and migration of T-lymphocytes into tumor sites. Additionally, on day 1 post-surgery the percentage of such patients remained unchanged, whereas on day 3 to 5 the number of patients amounted to 55% and remained as much as 40% on day 7 to 10 (Figure 3a–c). It should be noted that as much as 20% of patients showed the inflammation signs throughout the study (before surgery and close to the day of discharge; Figure 3b), while in 35% of patients the inflammation markers were registered from day 3 to day 10 post surgery (Figure 3c). The inflammation was inhibited and activated T-cell migration was balanced in 5% of patients only (not showed in Figure). Secondly, similarly to the first case, in 25% of patients signs of initial immune exhaustion and its functional activity suppression were observed in unchanged amount of 30% throughout the period post-surgery up to the day of patient discharge (Figure 3d), while in 25% these signs appeared only post-surgery and remained unchanged to the end of the study (Figure 3e). The immune system was normalized, and its functional activity was balanced by the end of the study in 15% of the patients only (Figure 3e).

**Figure 4:**
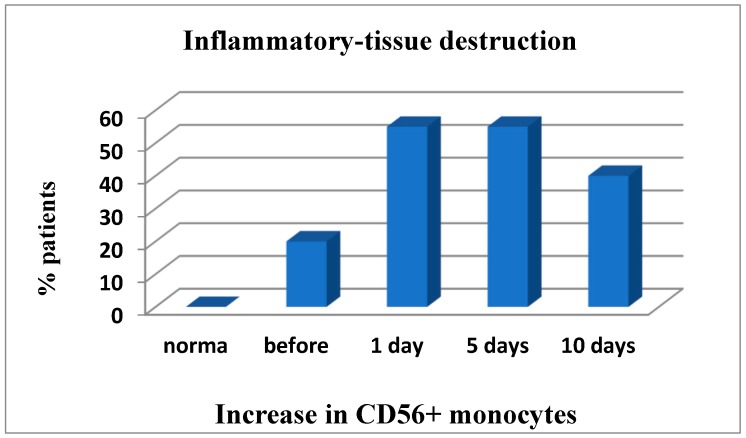
The development of inflammatory-destructive tissue damage in patients reflects an increase in CD56 + Mn. Number of days = after operation.

However, in 20% of patients inflammatory tissue-damaging processes (increased CD56+ monocytes (Figure 4); Patient depletion of the immune system—evaluation performed to raise and lower immune indexes) were recorded prior to surgery, but on day 1 to 5 post surgery the inflammation developed further and was already reported in 55% of patients and remained severe in 40% of patients by the end of the study (Figure 4).

Thus, the results are supportive of visible multifaceted changes in immune status following cryosurgery for pancreatic cancer. The number of clinical outcomes confirmed that the state of the immune system before cryosurgery was often a decisive factor to show if any disorders could occur post-surgery, or by contrast, if the immune system could be quickly normalized.

**Figure 5:**
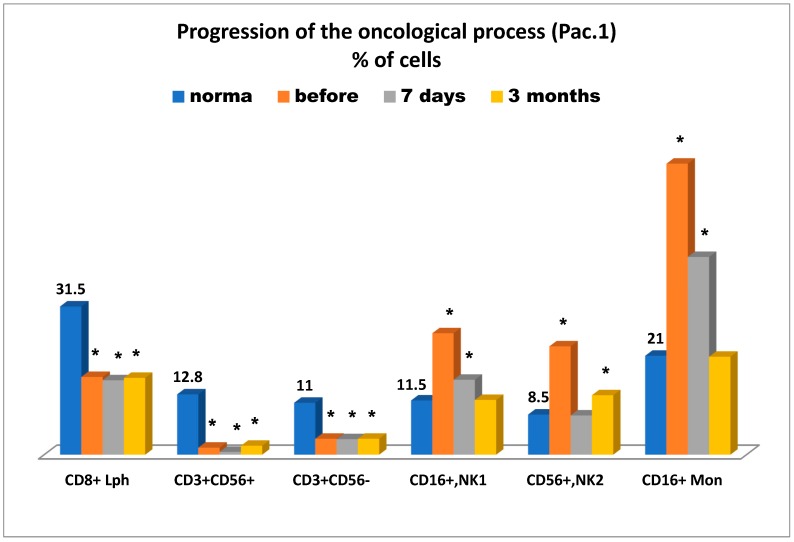
Progression of the oncological process in Patient 1; percentage of cells. Legend: * significance of differences; Lph = lymphocytes; Mon = monocytes; NK = natural killer cells; CD8 = cytotoxic T8-Lph; CD3+CD56+ = natural killer effector; CD3+CD56 = natural killer regulator; CD16+ Mon = Monocyte K-cell; number of days and months = after operation.

Admitted patient 1 complained of severe pain syndrome (9 on a 1–10 linear analogue scale) and significant unintentional weight loss. After examination carcinoma of the head of the pancreas was revealed (Т4N × M0) with vascular involvement and pancreatic and portal hypertension developed. The patient underwent cryosurgery, which was carried out using the CRYO-MT unit. Adenocarcinoma was confirmed morphologically. A marked relief of pain syndrome was reported in the early phase of the postoperative period. However, the combined therapy did not clinically result in disease stabilization and in the time following it led to disease progression. Figure 5 presents the state of the antitumor immunity over time—before cryosurgery, on day 7, and three months after cryosurgery for this patient.

It is of importance to mention (Figure 5) that the tumor-bearing patient was reported to have significant deficiency in killer cytotoxic T lymphocytes (CD8+), natural killer cells as effector cells (CD56+CD16+), and as regulator cells (CD56+CD16-), and more importantly they had a sharp increase in killer K-cells Mon (CD16+), which in the case in question, can more likely be considered as unconventional anti-inflammatory monocytes, supposedly belonging to subpopulation M3 [16]. They can further be transformed into tissue non-classical macrophage subpopulation М2, supporting cancer cell proliferation on top of other key known characteristics of this subpopulation. This fourth immune parameter at least coincides with the other three parameters (see above), which are supportive of pancreatic cancer progression associated with suppression of anti-tumor immunity. It is of interest to note that severe deficiency in anti-tumor immunity markers (cytotoxic T lymphocytes, natural killer cells as effectors, and regulators) was observed even three months after cryosurgery, whereas elevated levels of CD56+ monocytes were reported as early as on day 7 after cryosurgery and normalized in three months. To some extent it can be assumed that the elevated levels of natural killer cells 1 (CD16+) and 2 (CD56+) prior to cryosurgery may be indicative of cancer progression, especially that elevated levels of CD16+ T lymphocytes were also reported on day 7 after cryosurgery and normalized three months afterward, whereas elevated levels of CD56+ killer cells remained unchanged even after three months. During the entire period of immunological testing, NK cellular imbalance, associated with cancer instability, was at least reported. Therefore, it can be concluded that all immune-related findings could be supportive of an unfavorable prognosis for this patient in connection with cancer progression.

The second case study concerned patient 2, who was admitted to our center due to T4N×M0 carcinoma of the head of the pancreas up to 8 cm in size that invaded the celiac axis, common hepatic artery, superior mesenteric vein, and portal vein. After biliary pre-stenting, the patient underwent cryosurgery, which was carried out using a CRYO-MT unit. Adenocarcinoma was confirmed morphologically.

**Figure 6:**
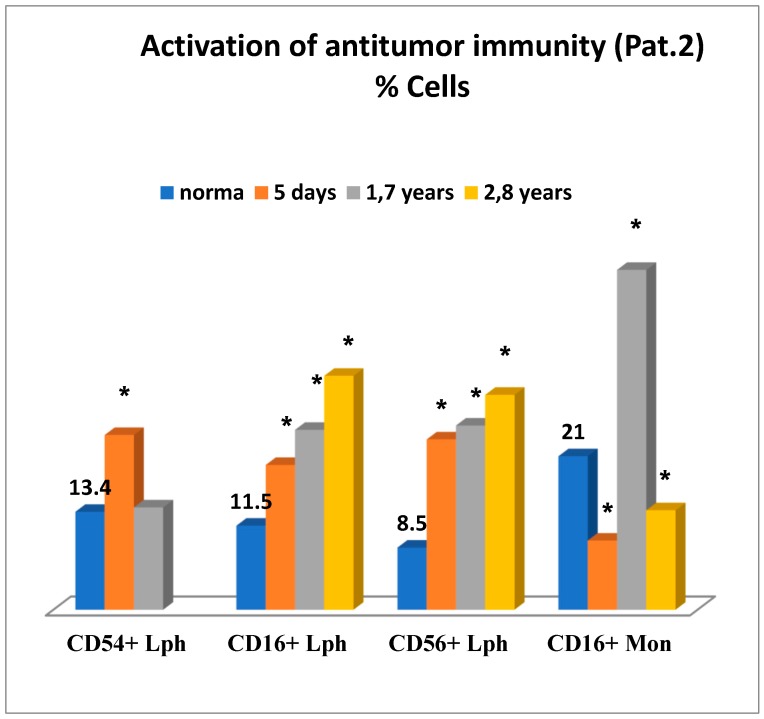
Activation of anti-tumor immunity in patient 2. Legend: * significance of differences; Lph = lymphocytes; Mon = monocytes; number of days = after operation.

**Figure 7:**
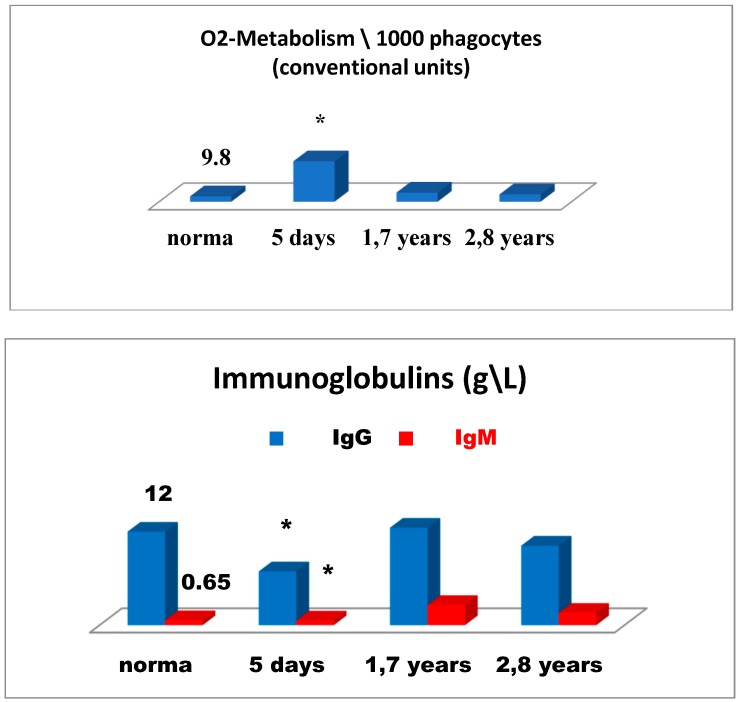
Oxygen metabolism of phagocytes and the level of immunoglobulins G and M in patient 2 in the early and late periods after cryodestruction of pancreatic cancer. Legend: * significance of differences.

Figure 6 is illustrative of a relatively successful treatment outcome and presents changes in the immune status for patient 2 in the early and remote stages after cryosurgery for carcinoma of the head of the pancreas. On day 5 after cryosurgery (Figure 6), activation of the immune system was mainly observed, which manifested itself as the evident elevation of lymphocytes expressing ICAM-1 (CD54+) adhesion molecules, known as ligands of LFA-1 (Lymphocyte Function-Associated Antigen 1) adhesion molecules, reflecting migration of lymphoid cells into tumor sites, and in the elevated levels of natural killer cells No. 1 (CD16+lymphocytes) and natural killer cells No. 2 (CD56+lymphocytes) and severe deficiency of killer K-cells monocytes (CD16+). Concurrently, humoral immune deficiency of IgG and IgM and acute phagocyte-derived oxidative stress were developed, which could have an adverse health outcome when affecting tissue metabolism in the body (Figure 7). 

Thus, on day 5 of the postoperative period, alternative changes in the immune system were observed altogether. On the one hand, possible activation of the anti-tumor immunity was initiated. On the other hand, changes were evident, such as possible impact on cancer progression, humoral immune deficiency development, and greatest oxidative stress as a possible tissue-damaging factor.

In order to minimize the risk of oxidative stress, to reduce postoperative inflammation at its site, and to enhance anti-tumor immunity, the combined immunotropic therapy was performed in the following dosage and administration: Reamberin (meglamune sodium succinate) was administered at a dose 400 mL intravenously very day in the course of five infusions. At day 2, 0.005% Imunofan (synthetic thymomimetic) was additionally introduced at a dose 1 mL intramuscularly every day in the course of seven injections. The postoperative period was uneventful, and the patient was discharged in satisfactory condition eight days after cryosurgery. Then, five courses of polychemotherapy were carried out according to GEMOX regimen (gemcitabine plus oxaliplatin). At a follow-up of 1.7 years and 2.8 years, the control immunological test results showed that the changes in the immune system reported earlier were normalized as compared to the previous test results. There was a reduction in the levels of lymphocytes expressing ICAM-1 (CD54+) adhesion molecules, in phagocyte-derived oxidative stress. There were no more signs of humoral immune deficiency (IgG, IgM), whereas anti-tumor immunity was significantly enhanced in connection with the elevation in different natural killer immune cells—NK-1, NK-2 (Figure 6 and 7). It is of importance to mention that at a follow-up of 1.7 years after cryosurgery (Figure 6), there was a sharp increase in the levels of CD16+ monocytes. However, in 2.8 years these levels were deficient, thus, proving the absence of cancer progression based on this marker, which could also be supportive of the indirect enhancement of the anti-tumor immunity. In connection with the ongoing chemotherapy, patient condition remained clinically stable at a follow-up of two years. According to ultrasound and MRA (magnetic resonance analysis) data, the following positive outcome was reported—the tumor was reduced in size by half to 4 × 4 cm. The patient-led an active lifestyle with good quality characteristics.

We can safely assume that cryosurgery in patients with unresectable pancreatic cancer is an intervention that considerably improves their quality of life, but primarily due to pain relief. Moreover, the obtained results suggest that cryosurgery is often a practical alternative. On the one hand, when used for pancreatic cancer cryosurgery induces activation of the immune system and anti-tumor immunity in particular, and concurrently it suppresses immune markers in the number of patients, on the other. Thus, based on the immune monitoring it becomes possible to predict the advisability of immunotropic therapy and its algorithm or completely discontinue its use at some treatment stage.

## 4. Conclusions

In conclusion, this article features two important phenomena that have been immunologically identified. (1) Some pancreatic cancer patients develop specific immune system disorders already at the preoperative stage. (2) In the postoperative period the disorders can either get worse or remain unchanged, their severity can be reduced, or they become evident only after cryosurgery. This becomes a prerequisite for affecting damaged immune targets and we remain hopeful that effective anticancer therapy will be found. Additionally, the immune system interventions can be carried out either before surgery and continued after it, or in the postoperative period when it is immunologically indicated, for as long in duration as is required, and in the presence of cancer recurrences. It is for this reason that the immune monitoring of patients is obligatory.

Cryosurgery must take a rightful place in pancreatic cancer therapy and should be considered as a reasonable alternative to existing types of surgery, or should be used as a component of multimodal therapy. The rationale for the combined use of topical cryosurgery, chemotherapy, and immunotropic therapy to increase anti-cancer therapy resistance and its capacity to eliminate tumors seems to be particularly promising.
